# Comparative Phytochemical Profile and Biological Activity of Four Major Medicinal Halophytes from Qassim Flora

**DOI:** 10.3390/plants10102208

**Published:** 2021-10-18

**Authors:** Hamdoon A. Mohammed, Hussein M. Ali, Kamal A. Qureshi, Mansour Alsharidah, Yasser I. Kandil, Rana Said, Salman A. A. Mohammed, Mohsen S. Al-Omar, Osamah Al Rugaie, Ahmed A. H. Abdellatif, Essam Abd-Elmoniem, Manal M. Abbas, Khalid M. Mohany, Riaz A. Khan

**Affiliations:** 1 Department of Medicinal Chemistry and Pharmacognosy, College of Pharmacy, Qassim University, Buraydah 51452, Saudi Arabia; 2 Department of Pharmacognosy, Faculty of Pharmacy, Al-Azhar University, Cairo 11371, Egypt; 3 Department of Pharmacology and Toxicology, College of Pharmacy, Qassim University, Buraydah 51452, Saudi Arabia; hu.ali@qu.edu.sa (H.M.A.); m.azmi@qu.edu.sa (S.A.A.M.); 4 Department of Biochemistry, Faculty of Medicine, Al-Azhar University, Assiut 71524, Egypt; 5 Department of Pharmaceutics, Unaizah College of Pharmacy, Qassim University, Unaizah 51911, Saudi Arabia; ka.qurishe@qu.edu.sa; 6 Department of Physiology, College of Medicine, Qassim University, Buraydah 51452, Saudi Arabia; malsharidah@qu.edu.sa; 7 Biochemistry and Molecular Biology Department, Faculty of Pharmacy, Al-Azhar University, Cairo 11371, Egypt; kandil.yasser@azhar.edu.eg; 8 Pharmacological and Diagnostic Research Centre, Faculty of Pharmacy, Al-Ahliyya Amman University, Amman 19328, Jordan; rana.said2@gmail.com; 9 Department of Medicinal Chemistry and Pharmacognosy, Faculty of Pharmacy, JUST, Irbid 22110, Jordan; m.omar@qu.edu.sa; 10 Department of Basic Medical Sciences, College of Medicine and Medical Sciences, Qassim University, Unaizah 51911, Saudi Arabia; o.alrugaie@qu.edu.sa; 11 Department of Pharmaceutics, College of Pharmacy, Qassim University, Buraydah 51452, Saudi Arabia; a.abdellatif@qu.edu.sa; 12 Department of Pharmaceutics and Industrial Pharmacy, Faculty of Pharmacy, Al-Azhar University, Assiut 71524, Egypt; 13 Department of Plant Production and Protection, College of Agriculture and Veterinary Medicine, Qassim University, Buraydah 51452, Saudi Arabia; ruessam2@yahoo.com; 14 Department of Medical Laboratory Sciences, Faculty of Allied Medical Sciences, Al-Ahliyya Amman University, Amman 19328, Jordan; m.abbas2@ammanu.edu.jo; 15 Department of Medical Biochemistry and Molecular Biology, Faculty of Medicine, Assiut University, El Fateh 71515, Egypt; khalidmohany@aun.edu.eg

**Keywords:** *Lycium shawii*, *Anabasis articulata*, *Rumex vesicarius*, *Zilla spinosa*, anticancer, antimicrobial, antioxidant, biogenetic interrelationship, flavonoid contents, trace elements

## Abstract

Four halophytic plants, *Lycium shawii*, *Anabasis articulata*, *Rumex vesicarius*, and *Zilla spinosa*, growing in the central Qassim area, Saudi Arabia, were phytochemically and biologically investigated. Their hydroalcoholic extracts’ UPLC-ESIQ-TOF analyses demonstrated the presence of 44 compounds of phenolic acids, flavonoids, saponins, carbohydrates, and fatty acids chemical classes. Among all the plants’ extracts, *L. shawii* showed the highest quantities of total phenolics, and flavonoids contents (52.72 and 13.01 mg/gm of the gallic acid and quercetin equivalents, respectively), along with the antioxidant activity in the TAA (total antioxidant activity), FRAP (ferric reducing antioxidant power), and DPPH-SA (2,2-diphenyl-1-picryl-hydrazyl-scavenging activity) assays with 25.6, 56.68, and 19.76 mg/gm, respectively, as Trolox equivalents. The hydroalcoholic extract of the *L. shawii* also demonstrated the best chelating activity at 21.84 mg/gm EDTA equivalents. Among all the four halophytes, the hydroalcoholic extract of *L. shawii* exhibited the highest antiproliferative activity against MCF7 and K562 cell lines with IC_50_ values at 194.5 µg/mL and 464.9 µg/mL, respectively. The hydroalcoholic extract of *A. articulata* demonstrated better cytotoxic activity amongst all the tested plants’ extracts against the human pancreatic cancer cell lines (PANC1) with an IC_50_ value of 998.5 µg/mL. The *L. shawii* induced apoptosis in the MCF7 cell lines, and the percentage of the necrotic cells changed to 28.1% and 36.5% for the IC_50_ and double-IC_50_ values at 22.9% compared with the untreated groups. The hydroalcoholic extract of *L. shawii* showed substantial antibacterial activity against *Bacillus cereus* ATCC 10876 with a MIC value of 12.5 mg/mL. By contrast, the *A. articulata* and *Z. spinosa* exhibited antifungal activities against *Aspergillus niger* ATCC 6275 with MIC values at 12.5 and 50 mg/mL, respectively. These findings suggested that the *L. shawii* is a potential halophyte with remarkable biological properties, attributed to its contents of phenolics and flavonoid classes of compounds in its extract.

## 1. Introduction

Secondary metabolite-derived compounds from plants serve the basic needs of humans and animals as medicaments [1,2,3]. Natural products have global acceptability and use due to their diversity, ease of access, sustainability, procurements, efficacy, safety, and widespread occurrence [4]. Nevertheless, some plants have been identified as toxic botanicals, including digitalis, belladonna, and ephedra, etc. [5]. However, these plants are widely used for specific purposes by people to treat certain critical disorders [6,7,8]. Since ancient times, the necessity to explore plants’ activity against various diseases has remained continued, and the approaches to the scientific confirmations of phytochemicals’ biological activity are a well-established tactic for new drug discovery, and drug development in modern times [6,9,10].

The environmental factors, and habitat-related effects, associated with high salinity, desert climate, and water and nutrient scarcity have emphasized the importance of halophytic plants in the fields of drug discovery, and alternative medicines [11,12]. These harsh environmental conditions compel plants to maintain higher levels of compounds with defensive roles which are produced as part of their survival mechanism against excessive oxidative stress, bacterial infection, and animal grazing encroachments [13]. Secondary metabolites, such as phenolics, flavonoids, alkaloids, and saponins comparatively higher presence make these plants more intriguing for chemical and biological evaluations [14,15,16]. Additionally, the presence of these compounds also confer significant nutritional and health-promoting benefits, including the medicinal properties in these plants [14,15].

Generally, the central region of Saudi Arabia has a high-salinity ecosystem, which affects plants’ growth, and is a significant challenge behind the slowed development of agriculture in the area [17,18]. The potential of some of these plants of halophytic nature, such as *Lycium shawii, Anabasis articulata, Zilla spinosa*, and *Rumex vesicarius*, to flourish in this harsh habitat demonstrates their species’ capacity to adapt to and survive in unfavorable environmental conditions and makes these plants noticeable for their presence and traditional medicinal uses, including their uses in livestock feeds, and human nutrition. The environmental adaptation of these plants is based on the presence of certain enzymes, and their productions of enzymatic and non-enzymatic compounds to protect these plants against intercellular oxidative stress caused by dryness of the habitat’s atmosphere, water scarcity in the soil, and soil salinity [14,19]. These plants also produce phenolics and flavonoid compounds to neutralize the reactive oxygen species (ROS) as part of their antioxidant defense systems [13,20]. Locally, these plants are also used as livestock feed [21,22] and are employed to treat various medical conditions of the ailing population. *Lycium shawii* has a rich history of use in central Saudi Arabia for infection control [23], and for allergy treatments in Wadi Hagul, Egypt [21]. *Rumex vesicarius* is used as a diuretic and for treatments of GIT (Gastro Intestinal Tract) disorders, including dysentery and dyspepsia [23]. The plant is also part of the human diet [24]. In Algeria, the aerial parts of the *Anabasis articulata* are decocted and used as a remedy for diabetes [25]. The entire plant has been used to treat hypertension [26]. *Zilla spinosa* has a long history of treating urinary and gallbladder stones [27] in this area of the region and has a purgative action [26].

The four plants, i.e., *Lycium shawii, Anabasis articulata, Zilla spinosa*, and *Rumex vesicarius*, were selected based on defined criteria [28], specifically including their high distribution in the Qassim region, similar halophytic character, soil type, the environmental similarity of growing conditions, and their common folklore uses in different ailments. The current study investigated the phytochemical contents, anticancer, antimicrobial, and antioxidant activities of the hydroalcoholic extracts of these four major halophytic plants growing in central Saudi Arabia. The study aimed to provide a comparative chemical contents status, and antioxidant potentials of the plants’ extracts, together with their different biological activity levels’ as examined in the in vitro conditions.

## 2. Results and Discussion

### 2.1. Trace Elements Analysis

Trace elements have various functions in maintaining the general health status of living organisms. Consequently, any disturbances in the trace elements’ presence levels, by either deficiency or their increments up to the toxic levels due to systemic accumulation, forms the core of the lead causes for several pathological disorders in the biosystems. The trace elements deficiency may result from either a decrease in specific element’s intake, or an increase in the levels of these elements due to any biochemical, physiological, or environmental causes, whereby both may lead to impairment, regression, and higher activity of the biochemical pathways to increase the risks for diseases generation over the time [29,30].

The edible halophytes are a good natural source of trace elements for the local population [12,15,31]. The high contents of trace elements in these halophytic plants are also considered responsible for supplementing their antioxidants and supporting several other biological actions [32]. Magnesium (Mg) is one of the important trace elements which acts as a cofactor for about ~300 enzymes, including the regulation of blood glucose, protein synthesis, regulation of blood pressure, and functions of both nerves, and the muscles [33]. A previous study also demonstrated that magnesium decreases the risk of ischemic heart disease, caused by the reduced blood supply to cardiac muscles [34]. Among all the four plants, magnesium was measured at higher levels in *Anabasis articulata* and *Rumex vesicarius* (1272 ± 18.52 µg/kg and 1250 ± 20.0 µg/kg); however, its contents in the *Lycium shawii* (1163 ± 9.17 µg/kg), and *Zilla spinosa* (1191 ± 10.14 µg/kg) (Table 1) were measured in parallel to other halophytes found in this region. Another abundant element, Manganese (Mn), has important functions in the activating and synthesizing many enzymes, e.g., isomerases, ligases, transferases, hydrolases, pyruvate decarboxylase, arginase, glutamine synthetase, lysates, and oxidoreductases. It also has a role in regulating blood glucose levels, improving immunity, and mineralizing the bones [35]. Moreover, manganese also supports intracellular metabolic energy production, and protects the cells from free radicals led damages [36]. Among these four halophytes, the manganese was detected at the highest concentration in *Rumex vesicarius* at 96.03 ± 1.04 µg/kg levels, and the lowest concentration was found in *Lycium shawii* at 28.53 ± 0.42 µg/kg occurrence, as detected in their respective plants’ dry powders. Iron (Fe) is part of the structures of multiple proteins, including mitochondrial cytochrome enzymes responsible for energy production, several cellular functions, and also for cell differentiation [37]. In addition, iron is also a component of myoglobin and hemoglobin and functions as a carrier of oxygen [38]. *Anabasis articulata* contained the highest levels of iron concentrations at 243.33 ± 2.08 µg/kg, while the *Zilla spinosa* showed the lowest levels of iron at 93.47 ± 1.16 µg/kg concentrations from the respective plants’ dry powders. Copper (Cu) represents one of the fundamental trace elements, also part of the structures of several enzymes, i.e., tyrosinase, cytochrome oxidase [39], and the antioxidant superoxide dismutase [40]. In addition, it also forms a component of copper-containing ceruloplasmin protein that is associated with red blood cells formation, and its deficiency leads to the progression of anemia [41]. All the studied halophytic plants in the current work contained copper at concentrations ranging from 10.34 ± 2.11 µg/kg to 13.67 ± 0.50 µg/kg of the plants’ dry powders. *Anabasis articulata* showed the highest levels of copper at 13.67 ± 0.50 µg/kg, while *Lycium shawii* had the lowest concentration levels of copper at 10.34 ± 2.11 µg/kg of the plants’ powder. Zinc (Zn), associated with several molecular, cellular, metabolic, and immunological functions, including antioxidant, anti-inflammatory, and anti-apoptotic responses [42], was also detected in all four halophytes. The element is essential for normal spermatogenesis, taste sensation, and gastric enzymes secretions [43]. A previous study reported that zinc is related to insulin secretion, and it increases insulin sensitivity for the tissue, and provides an improved glucose utilization for the diabetic conditions [44]. Cobalt (Co), another element present in these plants, represents a key constituent of vitamin B12 [45], and is part of the structure of methyl malonyl-CoA-mutase enzyme, which has a role in amino acid [46] and purine, as well as, pyrimidine metabolisms [47]. 

Moreover, cobalt also helps to normalize blood glucose levels, and increases adiponectin secretion, consequently improving the conditions of diabetes mellitus, obesity, hypertension, and cardiovascular diseases, and thus helps to treat them [48,49]. Zinc and cobalt contents of the plants play important roles in the biological activity and enhancement of the general health of humans and animals. Zinc and cobalt were detected in all the four plants with the highest levels detected in the *Rumex vesicarius* at 109 ± 0.0 µg/kg and 18.0 ± 3.55 µg/kg concentrations, respectively, while the *Zilla spinosa* contained the lowest concentrations of these two elements at 10.52 ± 1.10 µg/kg and 12.23 ± 2.07 µg/kg levels of the dried plants’ powders, respectively (Table 1). The overall mineral contents in these four plants are supportive indications of the traditional uses of these plants as part of food, livestock feeds, nutritional supplements, and medicinal herbs. The higher levels of the trace elements in these plants is also an indication of the presence of these elements in higher concentrations in the Qassim soil, and the plants’ adaptation and intake of these elements for various purposes of the plants’ physiology, biomechanism, metabolism, defense, and environmental factors.

### 2.2. LC-MS Profiles of the Plants 

The LC-MS analyses of the plants’ hydroalcoholic extracts’ results are summarized in Table 2 (for details see the Supplementary File).

**Figure 1:**
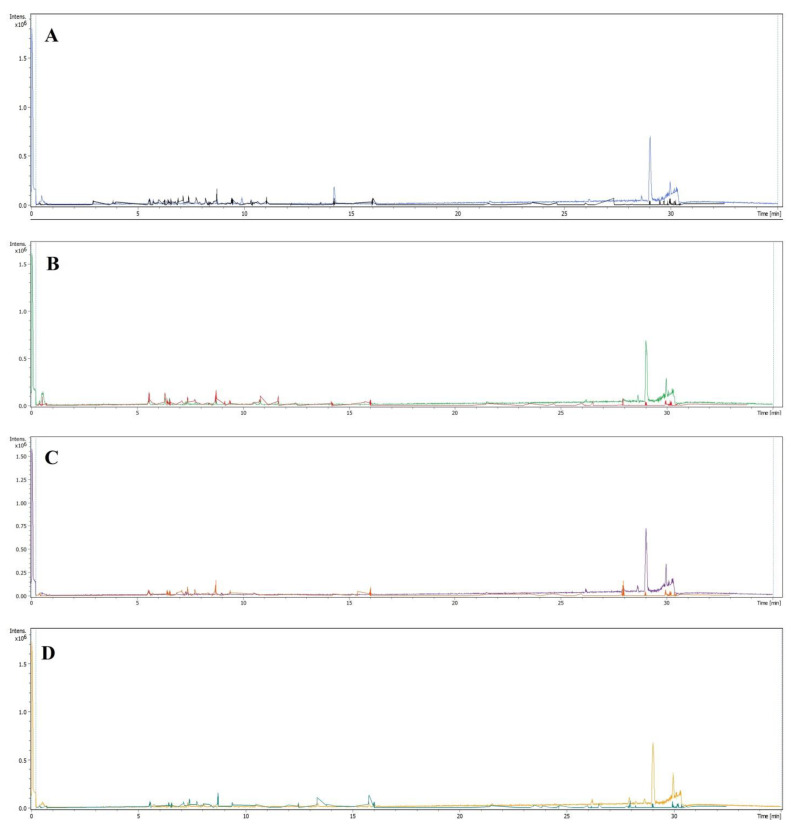
LC-MS chromatograms of the four halophytic plants; (**A**) *R. vesicarius*, (**B**) *L. shawii*, (**C**) *A. articulata*, and (**D**) *Z. spinosa*.

The tentatively identified compounds were arranged in an ascending elution order of the chromatographic analysis with the specific retention time of each compound. The compounds’ structures were tentatively assigned based on the mass spectral pattern, fragment ions peaks, and their abundances in the corresponding MS spectrum. The relative percentage of the identified compounds’ presence was calculated by considering their peak area concerning the area of all the peaks of the chromatogram. Forty-four compounds were identified, representing the primary and secondary plant metabolites, e.g., carbohydrates as sorbitol, galabiose, and hexose-based disaccharide. The presence of secondary metabolites, e.g., flavonoids, saponins, and phenolic acids were also confirmed. The LC-MS analyses results (Table 2) showed certain levels of similarities between the four halophytic plants’ constituents, which were detected in different proportions. For instance, the compound at a retention time of 5.6 min with the corresponding molecular weight (MW) at m/z 609.1427 [M-H] was tentatively identified as quercetin-3-glucosyl-7-rhamnosyl, which was detected in all the four plants in different concentrations but at the same retention time (Figure 1 and Table 2). Some fatty acids, i.e., oleic, linoleic, palmitic, and stearic acids, were also commonly present in all these plants, together with other long-chain saturated and unsaturated fatty acids. Only 27 of the 44 compounds identified in the current analyses were detected in *A. articulata* and *Z. spinosa*. However, the majority of the identified compounds were found in the *R. vesicarius* and *L. shawii* extracts, which were at 38 and 43 compounds, respectively, as found populated in the extracts through the LC-MS analysis.

Chromatographic analyses also exhibited that the total percentages of the identified compounds from each plant were 30.07 %, 21.99 %, 19.15 %, and 22.41 % for *R. vesicarius*, *L. shawii*, *A. articulata*, and *Z. spinosa*, respectively, based on the compounds’ discernible peaks as available in their respective LC-chromatograms. The presence of these compounds in the plants also revealed their health benefits as saccharides and fatty acids, together with the phenolics and flavonoids classes of compounds, which were present in abundance in these plants’ extracts. The presence of the saturated fatty acids, e.g., stearic acid, at higher proportions in the extracts of all the plants (from 6% to 11% of the identified compounds), is noticeable from the health-benefit view-point that makes the part of the human diet, probably also as the thrombogenic and atherogenic risk factors improvement entity, as reported earlier that the intake of 19 g of the stearic acid in the diet is effective [50]. The four plants also contained variable amounts of other dietary fatty acid constituents, i.e., oleic, palmitic, and linoleic acids, which are dominant in the halophytes [51,52] and are known to play roles with their nutritive values. Moreover, the highest percentages of the fatty acids were found in the *Z. spinosa* in comparison to other plants under the current study. In addition, sorbitol, hexose-based disaccharide, and L-acetyl carnitine were also identified in the extracts of all four plants at variable concentrations. The nutritional and medicinal values of these plants were also demonstrated by the presence of γ-tocotrienol, and gingerol in their extracts (Table 2).

**Figure 2:**
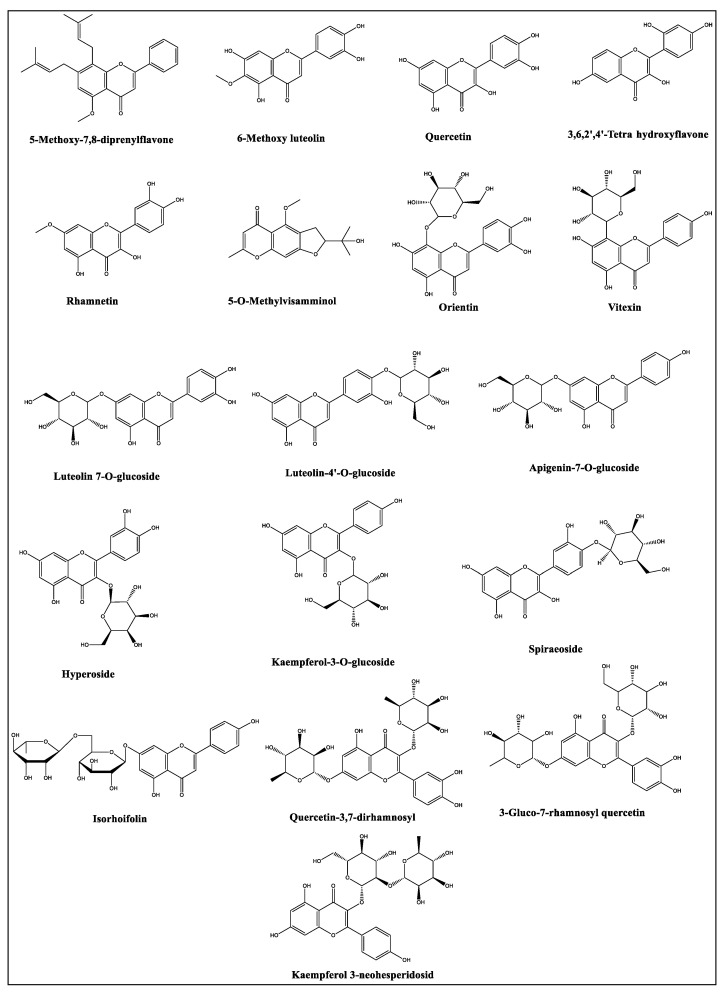
Structures of the flavonoid contents of the four halophytic plants.

Secondary metabolites, flavonoids, and phenolics are also reported for their health benefits [53,54] and are known to possess pharmacological activities of different kinds, including antimicrobial [55,56], anticancer [55,57,58], and several other [59,60,61]. The results in Table 2 showed noticeable variations in the phenolics and flavonoids distributions among these investigated four plants. Among the flavonoids and phenolics compounds identified in the plants under current investigation, hyperoside, quercetin, apigenin-7-O-glucoside, rhamnetin, and chlorogenic acid [62,63,64,65] have been reported for their antimicrobial activity, whereas spiraeoside, orientin, luteolin-7-O-glucoside, vitexin, isovitexin, quercetin, and apigenin-7-O-glucoside [62,63,64,66,67,68,69] have been reported for their anticancer activities. The results in Table 2 also demonstrated the presence of three flavonoids, i.e., quercetin-3,7-dirhamnosyl, quercetin-3-glucosyl-7-rhamnosyl, and 3-O-neohesperidoside kaempferol, which were identified in all the four plants; however, their representative occurrence (percentages) in these plants varied. The highest number of identified flavonoids were represented in the *L. shawii* extract (18 compounds representing 3.20% of the total peaks area of the plant extract’s LC-MS chromatogram) followed by the *R. vesicarius* extract (16 compounds representing 3.34% of the total peaks area of the plant’s extract’s LC-MS chromatogram). The lowest numbers, and percentages of the flavonoids were identified in *A. articulata* and *Z. spinosa*, which showed eight (0.0431% of the area of all the peaks of the plant’s extract’s LC-MS chromatogram) and five (0.33% of the total area of all the peaks in the plant’s extract LC-MS chromatogram) of the identified flavonoids, respectively. The structures of the flavonoid contents, varied in C_6_-C_3_-C_6_ flavonoid basic skeletal substitutions of hydroxyl, methylation, prenylation, and glycosylations of mono- and di-glycosidic nature, were encountered; their structures are provided in Figure 2.

The reported biological activities of these plants and their common use in traditional medicine are mostly attributed, but maybe not limited, to their contents of flavonoids and phenolics compounds. Furthermore, the variations in the phenolics and flavonoids constituents’ representations, and concentrations could be among the major reasons for variations observed in their antioxidant, antimicrobial, and anticancer activities as encountered in these plants in the current study (Table 3, Table 4, Table 5 and Table 6). The presence of major phenolics and flavonoid compounds also indicated the roles of the antioxidant compounds in these four plants in relation to exhibiting the combined antioxidant potential of each plant, and their treatment efficacy vis-a-vis their uses in containing, and curing of various disease, especially, of the oxidative stress origins. The complex flavonoids structures of mono- and di-glycosidal patterns have lent credence to the known roles and receptor interactions of flavonoids and their glycosides in various molecular modeling based studies [70]. This approach also established the attempt of comparison study of these four halophytes in relation to their constituents, and their biological activities.

### 2.3. Total Phenolic and Flavonoid Contents

The quantitative analysis of the total phenolics and flavonoids were also conducted since the percentages of the identified constituents obtained by LC-MS analysis only showed 30.07%, 21.99%, 19.15%, and 22.41% as the identified constituents in these plants, which meant that the percentages of the unidentified constituents by the LC-MS technique were at 69.92%, 78.01%, 80.85%, and 77.59% of the plants’ constituents from these four plants, i.e., *R. vesicarius*, *L. shawii*, *A. articulata*, and *Z. spinosa*, respectively (Table 2). Although, all the four plants under investigation are growing in a similar environment and locality with similar salinity levels and in the marshy area, the results in Table 3 for phenolics and flavonoid quantities revealed distinct variations among these plants’ species. For instance, the phenolics contents in *A. articulata*, and *Z. spinosa* were at the lowest levels of 21.13 mg/gm and 22.36 mg/gm GAE (Gallic Acid Equivalent) as compared to the *R. vesicarius* contents at 28.54 mg/gm GAE, respectively, of the dried extracts of the respective plants. In addition, nearly two folds of the *R. vesicarius*, and more than two-folds of the *A. articulata* and *Z. spinosa* phenolics contents levels, the phenolics contents were measured in the *L. shawii* extract which was found at 52.72 mg/gm of the GAE of the dried plants’ extracts. Despite these differences in the phenolics contents among these species, the assays revealed that the flavonoids quantity in three out of these four plant species, i.e., *L. shawii, A. articulata*, and *R. vesicarius*, were at higher levels of 11.48 to 13.01 mg/gm QE (Quercetin Equivalent) of the dry extracts of the respective plants. By contrast, *Z. spinosa* extract accounted for the lowest flavonoids contents at 7.29 mg/gm QE of the plant’s dried extract. The results obtained for the phenolics and flavonoids contents analysis in these species were consistent with the LC-MS chromatographic profiling of the respective plants, and they exhibited similar patterns of the presence of these constituents which validated the current findings. For instance, the highest numbers of the identified phenolics and flavonoids by the LC-MS were recorded for the *L. shawii* extract (18 flavonoids representing 3.20 % of the total peaks’ area of the chromatogram), and *R. vesicarius* (16 compounds representing 3.34 % of the area of all the peaks of the chromatogram). The lowest identified phenolics and flavonoid contents were observed in the *A. articulata*, and *Z. spinosa* (8 compounds, 0.0431%, and 5 compounds, 0.33%, respectively, of the total peaks areas of their respective chromatograms).

The influence of environmental conditions on the plant constituents can be postulated by comparing the levels of phenolics and flavonoids contents in *R. vesicarius* against the similar species of the plant growing in Algeria that contained 43.28 and 19.72 mg/gm catechin equivalents of phenolics and flavonoids contents, respectively [71]. However, for the plant species growing under similar conditions, the total phenolics and flavonoid contents in *L. shawii* revealed 101.70 mg/gm GAE and 59.8 mg/gm of QE of the phenolics and flavonoids, respectively [72]. The current levels of *Z. spinosa* phenolics and flavonoids contents were nearly similar to that recorded for the species growing in the southern part of Saudi Arabia [73]. The presence of phenolics and flavonoid contents in these plants also supported the use of these plants as part of foods and livestock feed, as well as their use for different biological activities where the roles of antioxidants are involved [74,75,76].

### 2.4. Antioxidant Activity

The antioxidant potentials of the plants were measured with different methods to evaluate the free radicals scavenging potentials (2,2-diphenyl-1-picrylhydrazyl-scavenging activity, DPPH-SA), metal chelation potentials (metal chelating activity, MCA), and reducing-power of the plants’ extracts (total antioxidant power, TAP, and ferric reducing antioxidant power, FRAP). The antioxidant estimations results (Table 3) confirmed the positive relationship between the antioxidant potentials of the plants and their phenolics and flavonoids contents. The *L. shawii* extract contained the highest phenolics and flavonoids contents, and the highest level of antioxidant activity was observed for this plant as compared to all the other plants investigated under the current study. The extract of *L. shawii* reduced the molybdenum (VI) to molybdenum (V) in the TAA, and ferric ions in the FRAP estimation by 26.60 mg and 56.68 mg of Trolox equivalent (TE), respectively. The next higher levels of reducing activity were recorded for *R. vesicarius*, followed by *Z. spinosa*, and *A. articulata* with FRAP activities equal to 33.09, 23.68, and 19.67 TE/gm of the plants’ extracts. The TAA of *R. vesicarius* was less than the *Z. spinosa* and *A. articulata*, thereby suggesting that these extracts reduced the ferric ions more than the molybdenum (VI) ions as compared between the two methods results. The scavenging activity of *L. shawii* extract against DPPH free radicals scavenging (19.76 TE) was significantly higher than those of the other plants’ extracts. However, the *R. vesicarius* DPPH-SA (14.22 TE) activity was as high as twice to that of the *Z. spinosa*, and *A. articulata* plants at 7.22 and 7.15 TE, respectively. The results displayed in Table 3 also confirmed higher iron-chelating power of the extracts of *L. shawii* of MCA at 21.84 mg EDTA-equivalents, and *R. vesicarius* and *Z. spinosa* showed the MCA at 13.01 mg and 13.32 mg of EDTA-equivalents, respectively. A lower MCA potential was recorded for *A. articulata* extract. The overall antioxidant potential of all the four plants revealed that the phenolics and flavonoid contents have an essential role in these plants’ antioxidant capacity.

### 2.5. Cytotoxicity Analysis

**Figure 3:**
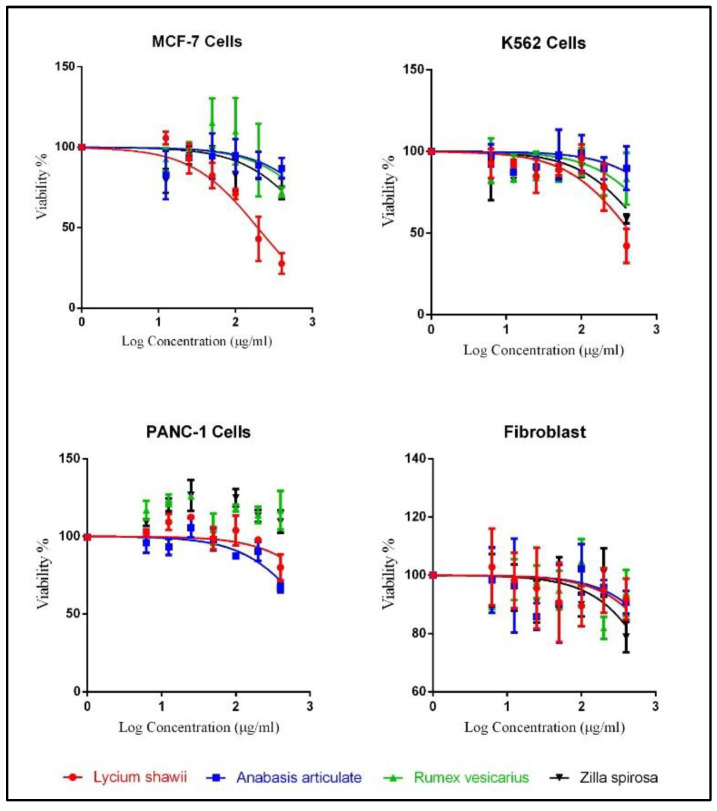
The log dose-response curve of cancer cell lines compared to the fibroblasts. Values are represented as mean ± SD.

The cytotoxic activity evaluations were conducted for all the plants’ hydroalcoholic extracts’ against the three cancer cell lines, i.e., breast cancer cell line (MCF-7), chronic myeloid leukemia cell line (K562), and the human pancreatic cancer cell line (PANC-1) (Supplementary File). The fibroblast cell lines were also cultured with the plant extracts to measure their toxicity on the normal cells. Among all the four halophytes, the hydroalcoholic extract of *L. shawii* showed the highest antiproliferative effects against MCF-7 and K562 cell lines with IC_50_ values at 194.5 µg/mL and 464.9 µg/mL, respectively. The hydroalcoholic extract of *A. articulata* demonstrated the highest cytotoxic activity against the human pancreatic cancer cell line (PANC-1) with IC_50_ values at 998.5 µg/mL (Table 4). The plant extracts’ cytotoxic effects against MCF-7 and K562 cell lines were dose-dependent (Figure 3). The results in Table 4 also demonstrated the plants’ safety toward the normal fibroblast cells, as the IC_50_ were measured above 3000 µg/mL for all the plants’ hydroalcoholic extracts, except for the *Z spinosa* hydroalcoholic extract, which showed an IC_50_ value at 1888 µg/mL. The overall results indicated a higher selectivity of *L. shawii* extract towards the MCF-7 and K562 cell lines as compared to the PANC-1 cell lines, and the fibroblast’s normal cell lines.

**Figure 4:**
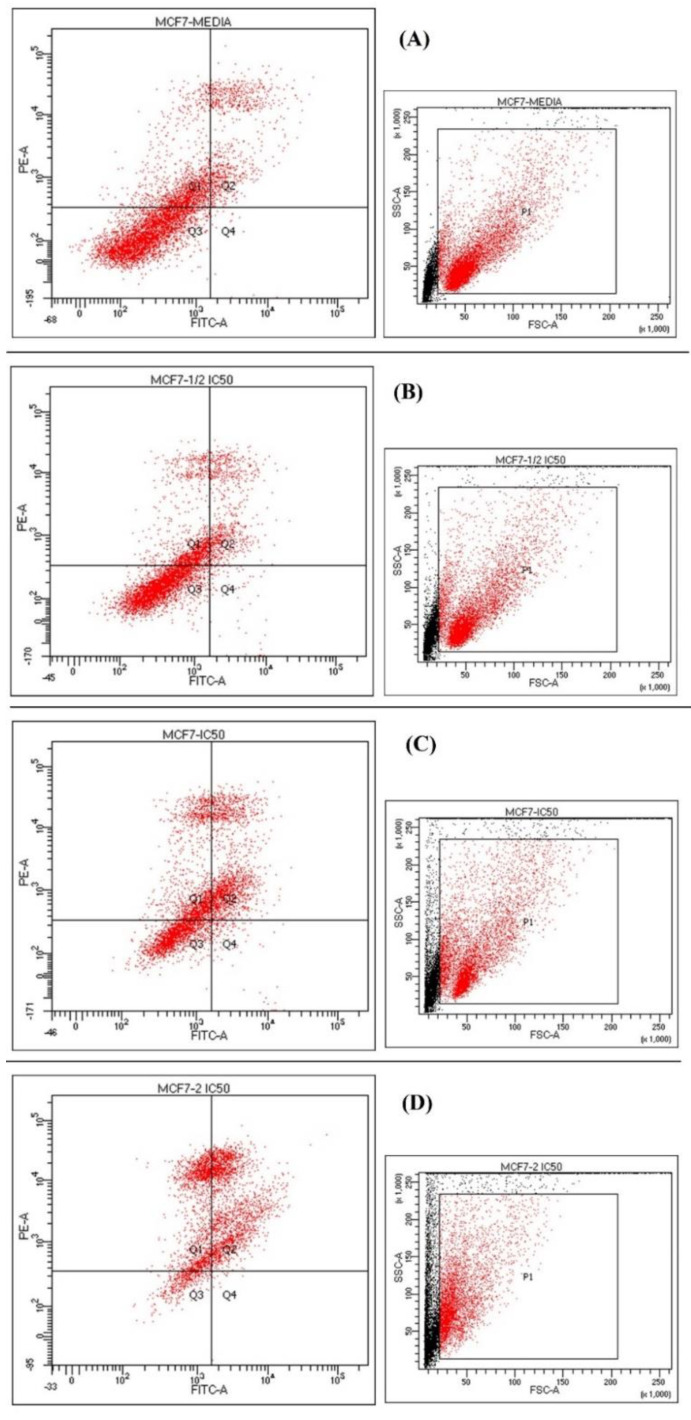
Effects of *L. shawii* extract treatment on apoptosis in MCF-7 cell lines. (**A**) Untreated control, (**B**) 0.5 % DMSO, (**C**) IC_50_ of *L. shawii* extract (519.2 μg/mL), and (**D**) double IC_50_ of *L. shawii* extract (1038.4 μg/mL).

The flow cytometry analysis demonstrated that the *L. shawii* could induce apoptosis in MCF-7 cell lines (Figure 4). Annexin-V conjugated-FITC represented the apoptotic cells, while the PI dyes represented membrane damage due to the necrosis and late apoptosis. The untreated cells expressing the negative group viability were at 94%, while the viable cells decreased, for both the IC_50_ and double IC_50_, at 36.2% and 9.5% for the *L. shawii*. However, half IC_50_ did not show any significant changes. The necrotic cells percentage for *L. shawii* increased to 28.1% and 36.5% for the IC_50_ and 2 × IC_50_ as compared to the 22.9% of the untreated group (Table 5). In addition, using the concentrations equal to IC_50_ and 2 × IC_50_ for *L. shawii* extract, the late apoptotic cells viability increased to 33.4% and 53.6%, respectively. These results demonstrated that the *L. shawii* cytotoxic effects were dose-dependent, as, once the concentration was increased from IC_50_ to double IC_50_, the percentage of the necrosis increased in a directly proportional relationship. These data also confirmed that the *L. shawii* extract could inhibit the growths of human cancer cell lines, MCF-7, effectively.

### 2.6. Antimicrobial Activity

#### 2.6.1. Preliminary Antimicrobial Screening

**Figure 5:**
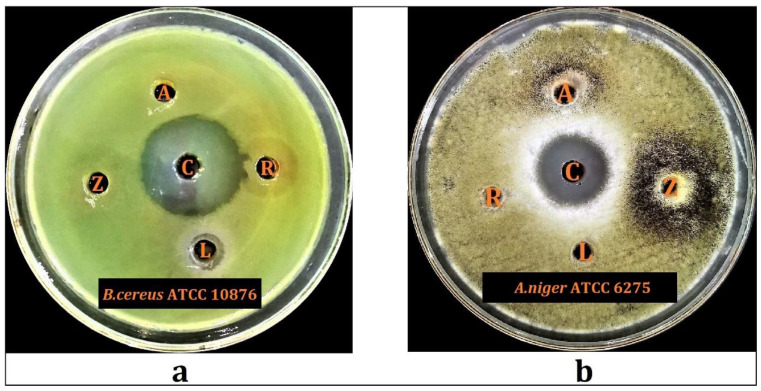
Results of preliminary antimicrobial activity evaluations against, (**a**) *B. cereus* 10876; (**b**) *A. niger* 6275 for *Lycium shawii* (**L**), *Anabasis articulata* (**A**), *Rumex vesicarius* (**R**), *Zilla spinosa* (**Z**) plants’ hydroalcoholic extracts, and control antibiotics (**C**).

Results of the preliminary antimicrobial screenings of the hydroalcoholic extracts of *L. shawii*, *A. articulata*, *R. vesicarius*, and *Z. spinosa* revealed that *L. shawii* possesses significant antimicrobial activity against the tested *Bacillus cereus* ATCC 10876, whereas the hydroalcoholic extracts of the *A. articulata* and *Z. spinosa* exhibited substantial antifungal activity against the tested *A. niger* ATCC 6275 (Figure 5). The results demonstrated that the hydroalcoholic extract of *L. shawii* inhibited *B. cereus* ATCC 10876 with a diameter of 9.0 ± 0.1 mm, while *A. articulata* and *Z. spinosa* inhibited *A. niger* ATCC 6275 with diameters of 7.5 ± 0.2 mm, and 7.7 ± 0.2 mm, respectively. Additionally, Table 6 also displayed that the *L. shawii*, *A. articulata*, *R. vesicarius*, and *Z. spinosa* have no antimicrobial activity against other tested strains, whereas *R. vesicarius* exhibited no antimicrobial potential against all of the tested microorganisms. The currently observed weak antimicrobial activity of some of these plants’ extracts in comparison to their reported antimicrobial activity, from other plants’ counterparts found in the non-halophytic environment, could be attributed to their environment and habitat’s variations effects on these plants, which also seemingly have affected their constituents in concentrations and types, which were produced as a result of the non-halophytic conditions of these plants. The desert climate effects on the anti-microbial activity of the currently studied four plants demonstrated the effects of the plant environment on the constituents and their biological activity. For example, *L. shawii* plant species growing in Yemen and Tunisia have been reported to have antimicrobial activity against several microbes, including, *S. aureus, K. pneumoniae*, and *E. faecalis*, whereas the *L. shawii* species growing here in this region of Saudi Arabia was found to be inactive against these microbes [77,78]. Moreover, the current findings for the antimicrobial activity of *L. shawii, A. articulata*, and *R. vesicarius* were consistent with the previous findings regarding the antimicrobial activity of these plant species growing in the similar environmental conditions in Saudi Arabia but at different locations than ours; these plants contained these activities [79].

#### 2.6.2. Minimum Inhibitory Concentration and Minimum Bacterial Concentration

The tests results indicated that the hydroalcoholic extract of *L. shawii* had MIC and MBC values of 12.5 mg/mL, and 25 mg/mL, respectively, against the tested *B. cereus* ATCC 10876. By comparison, the *A. articulata* exhibited MIC and MBC values of 12.5 mg/mL, and 25 mg/mL, respectively, against the *A. niger* ATCC 6275, whereas the *Z. spinosa* showed MIC and MBC values at 50 mg/mL, and 100 mg/mL, respectively, against *A. niger* ATCC 6275. At the same time, the control antibiotics inhibited the growths of all the tested organisms at the given concentrations. The results are summarized in Table 6.

### 2.7. Plants’ Antioxidant Potential, Biological Activity, Flavonoids Plausible Biogenetic Interrelationships, and Molecular Oxygen Proliferations

The current investigation on these four plants exhibited a discernible biogenetic interrelationship in the occurrence of different flavonoid molecules in each plant. The LC-MS analysis revealed that the polyhydroxylated flavonoid aglycones, i.e., quercetin, luteolin, and kaempferol, and their mono- and di-glycosides were the dominant flavonoids among these plants. The progressing oxygen proliferations with the advancing molecular weights in the series of flavonoids present in these plants’ extracts showed the increasing anti-oxidative effects of these plant extract in relation to their levels of the, primarily, the flavonoid contents.

**Figure 6:**
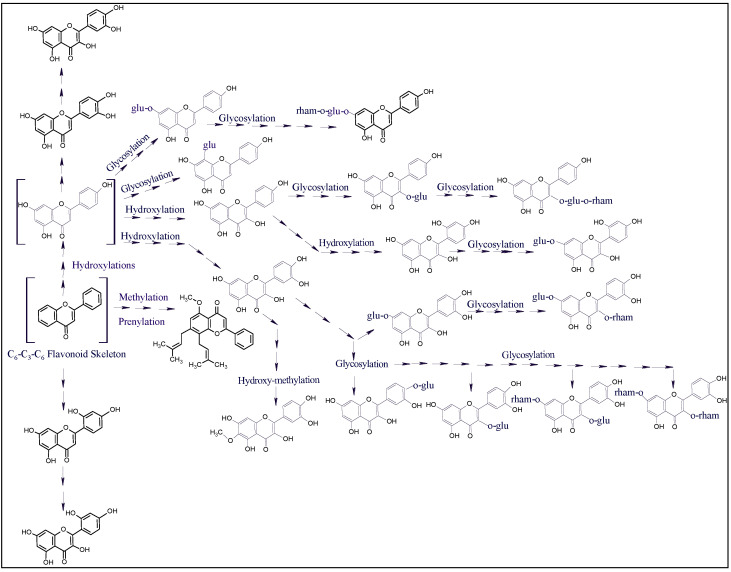
The proposed plausible biogenetic interrelationship and molecular oxygen proliferation in flavonoids from different plants. The products shown in parenthesis have not been detected in the current study.

**Figure 7:**
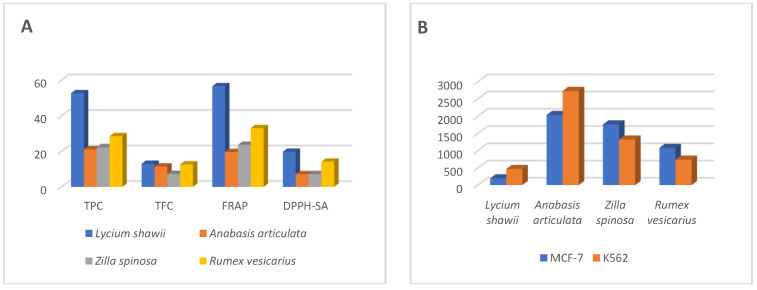
Comparative flavonoids and phenolics contents, antioxidant (**A**), and anticancers (**B**) activities.

The plant’s deducible antioxidant levels in relation to their proportions of increasing levels of the antioxidant compounds, especially, the flavonoid contents were observed in these plants which have been observed in the LC-MS analysis and the quantitative estimations of the flavonoid contents and the plant’s antioxidant potential, e.g., the *L. shawii* showed the strongest antioxidant activity with the highest concentrations of the phenolics and flavonoids, where 18 flavonoid constituents, highest in numbers and all detected, were present in *L. shawii*. Moreover, the lowest antioxidant level was recorded for the *A. articulata*, and *Z. spinosa*, which have the lowest concentrations of these products. Nonetheless, an observation in the increasing flavonoid contents as a factor of increasing molecular oxygen proliferations in the flavonoids (Figure 6) of these halophytic plants was also found associated with the efficacy of their biological activity, e.g., *L. shawii* showed the strongest anticancer activity (lowest IC_50_ value against MCF-7 and K562 cell lines, Figure 7). The results demonstrated that quantities of phenolics and flavonoids contents in the four plants directly correlate with the plants’ antioxidant and anticancer activity levels.

The molecular oxygen proliferations in the flavonoid framework biogenetically produced different, multiple typed, and advancing molecular weights flavonoid contents in alignment with the demands of the halophytic plants to meet their specific requirements of antioxidant potency, disease, and pathogens-fighting capability as part of their defense mechanism [12].

## 3. Materials and Methods

### 3.1. Plant Materials and Extractions

Aerial parts of the plants were collected in March 2019 from the Qassim University campus surrounding areas. The plants were identified by the institutional botanists at the Department of Plant Production and Protection, College of Agriculture, Qassim University. The plants were dried in shade at room temperature (25 ± 2 °C) for two weeks before grinding. Afterward, 300 gm of plant powders were extracted with 95 % *v*/*v* ethanol (hydroalcoholic mixture) (3 × 1 L) for 24 h in stirring conditions. The extracts obtained from each plant were double-filtered through filter papers, and evaporated to dryness on a vacuum rotatory evaporator, Rotavapor^®^, under a temperature below 40 °C to give a dry gummy mass. The hydroalcoholic plant extracts were stored at −80 °C till further use.

### 3.2. Liquid Chromatography-Mass Spectroscopy (LC-MS) Analysis

A Bruker Daltonics (Bremen, Germany) Impact II ESI-Q-TOF (electrospray ionization- quadrupole time-of-flight) system equipped with Bruker Daltonics Elute UPLC system (Bremen, Germany) was used for extracts’ scanning under 190 nm and 500 nm range. Specific standards were used to identify the analyte’s retention time in the chromatographic analysis. Accurately, 1 mg of the plants’ extracts were dissolved in 2.0 mL of DMSO (analytical grade), and the solutions were diluted with acetonitrile to 50 mL. The obtained solutions were centrifuged at 4000 rpm for 2.0 min, 1.0 mL of the clear extracts’ solutions were transferred to the autosampler, and the injection volume was adjusted at 3.0 µL. The instrument was operated using Ion Source Apollo II ion Funnel electrospray source. The instrument parameters were adjusted as capillary voltage (2500 V), nebulizer gas (2.0 bar), nitrogen flow (8 L/min), and dry temperature (200 °C). The mass accuracy was 0.1 Da, the mass resolution was 50,000 FSR (full sensitivity resolution), and the TOF repetition rate was up to 20 kHz. Chromatographic separation was performed on 120, C_18_ reverse-phase (RP) column, 100 × 2.1 mm, 1.8 µm (120 Å) from Bruker Daltonics (Bremen, Germany) at 30 °C, and autosampler temperature at 8 °C with a total run time of 35 min using the gradient elutions. The eluent A and B consisted of methanol/5 mM ammonium formate/0.1 % formic acid, and water/methanol (90:10)/5 mM ammonium formate/0.1 % *v*/*v* formic acid, respectively.

### 3.3. Quantitative Measurements of the Total Phenolics and Flavonoids Contents

The method of Quy [80] was used for the measurement of total phenolics and flavonoids contents in the plants’ extracts as equivalents to gallic acid and quercetin using Folin–Ciocalteu, and aluminum chloride reagents, respectively. For the phenolics quantification, 0.2 mL of the 10 % *w*/*v* sodium carbonate solution was mixed with 1.6 mL of each plant extract (0.1 mg/mL), and 0.2 mL of the diluted Folin–Ciocalteu reagent (1:5 in distilled water). The mixture was vigorously mixed and kept for 30 min at RT before the absorbances were measured at 760 nm. Three independent measurements were recorded, and total phenolics contents of the plants’ extracts were expressed as gallic acid equivalent (GAE) per gm of the dried extract using the slope equation of the gallic acid calibration curve. The total flavonoids were measured by mixing 2 mL of the extracts (0.1 mg/mL), and 0.1 mL of the aluminum chloride (10 % in distilled water) with 0.1 mL of the potassium acetate (0.1 mM) in a test tube. The mixture’s absorbance was measured after 30 min incubation at 415 nm, and the quantified total flavonoids were expressed as quercetin equivalent (QE) per gm of the dried extract from three consecutive measurements.

### 3.4. Trace Elements Analysis

The dried plants’ powder was used to determine Fe, Cu, Mn, Co, Mg, and Zn trace elements’ presence using ICP-OES (Model iCAP 7400 Duo, serial IC 74DC144208, China) instrument according to the reported method of Johnsson [81]. The plants were dried at 70 °C for two days and sifted through a stainless-steel mill < 5 mm pore size. The dried plants’ materials (0.5 g) were digested in a mixture of strong acids, including HNO_3_, HCIO_4_, and H_2_SO_4_ (7:2:l) [12], and the trace elements’ concentrations were measured from the calibration curve prepared for the individual standard elements. The measurements were conducted in triplicate and were expressed as the mean of the results with their standard deviations.

### 3.5. Antioxidant Activity

#### 3.5.1. Total Antioxidant Capacity

The plant extracts’ antioxidant capacity was measured using the method described by Arwa et al. [82]. The molybdate reagent was prepared by mixing sulfuric acid (0.6 M), and ammonium molybdate (4 mM) in sodium phosphate buffer (28 mM). Accurately, 3.6 mL of the molybdate reagent was added to 0.4 mL of the plant extract (containing 200 µg of the extract), and the mixture was vortexed and kept in a warm water bath for 30 min. The mixture was allowed to cool at room temperature, and the absorbance was recorded at 695 nm using a spectrophotometer against a blank, which was prepared in a similar way by mixing 0.4 mL of distilled water with a molybdate reagent. The total antioxidant activity of the extract was calculated as the equivalent of the Trolox using the standard calibration curve from the following equation:

y = 0.1954x − 0.1788; R² = 0.9646

where y is the absorbance of the sample at 695 nm, and x is the concentration of the sample in µg/mL.

#### 3.5.2. DPPH Scavenging Activity

The ability of plant extracts to scavenge the DPPH-free radicals was determined as Trolox equivalents according to the method of Shimada et al. [83]. In brief, 1 mL of the extract’s solution in methanol (containing 200 µg of the extract) was added to 1 mL of the DPPH (prepared by dissolving 6 mg of the DPPH in 50 mL of methanol). The mixture was vortexed and kept standing for 30 min in the dark at room temperature. The absorbance of the mixture was measured at 517 nm by a spectrophotometer against methanol as a blank. The method was conducted in triplicate, a standard calibration curve of the Trolox against DPPH was prepared, and the Trolox equivalence of the extract was calculated from the curve slope equation.

#### 3.5.3. Ferric Reducing Antioxidant Power (FRAP) Assay

The FRAP assay was conducted by the method of Benzie and Strain [84] with minor modifications. The working reagent of the FRAP was freshly prepared by mixing one-fold of the TPTZ (2,4,6-Tris(2-pyridyl)-s-triazine, 10 mM prepared in 40 mM HCl), one-fold of the FeCl_3_·6H_2_O (20 mM), and ten-folds of acetate buffer (300 mM, pH 3.6). Accurately, 2 mL of the FRAP reagent was added to 0.1 mL of the extract (containing 200 µg of the dried extract), the mixture was incubated for 30 min at room temperature, and the absorbance was recorded at 593 nm. The procedure was conducted in triplicate, and the prepared FRAP-Trolox calibration curve was used to calculate the extract activity as mg Trolox equivalent per gm of the plant’s dried extract.

#### 3.5.4. Metal Chelating Activity Assay

The plant’s hydroalcoholic extract’s ability to chelate metals compared to the EDTA was estimated using the method of Zengin et al. [85]. In brief, a mixture of the extract solution (2 mL of ethanol containing 200 µg of extract) and ferrous chloride (25 µL, 2 mM) was added to 100 µL of ferrozine to inchoate the color. The mixture’s absorbance was recorded at 562 nm against a blank (2 mL of the extract plus 200 µL of the ferrous chloride without ferrozine). The standard calibration curve of EDTA was prepared, and the chelating activity of the extract was calculated as the equivalent of the EDTA.

### 3.6. Cytotoxic Assay

Extracts of *L. shawii, R. vesicarius, Z. spinosa*, and *A. articulata* were tested for their antiproliferative activity. Toxicity of all the extracts was measured against normal human fibroblast, MCF7, PANC-1, and K562 cell lines using standard MTT assay (Promega, Madison, WI, USA), which measured the ability of the mitochondrial dehydrogenase to reduce MTT to a purple formazan product. The cells were suspended at a density of 12–15 × 10^3^ cells/mL in RPMI 1640 media, and 100 µL of each cell type were seeded in each well of a 96-well microtiter plate and incubated for 24 h. The extract was dissolved in DMSO and added to the wells in triplicate to a final concentration ranging from 400 to 12.5 µg/mL in a 2-folds serial dilution (400, 200, 100, 50, 25, and 12.5 µg/mL), and incubated at 37 °C, 5% CO_2_ for 24 h. Two controls were used: one contained medium with cells, and the other contained cells plus medium with the vehicle. In addition, the extract was added without cells to check the effects of the background colors. Doxorubicin was used as a positive control. The tests were performed according to the manufacturer’s guidelines, and the absorbance was measured at 590 nm using a microplate reader (Biotech, Washington, DC, USA).

### 3.7. Apoptotic Assay

MCF7 cell lines (5 × 10^4^/well) were plated in 6-well plates 24 h before the experiment. Cells were treated with the inhibitory 1/2 (IC_50_), (IC_50_), and double (2xIC_50_) of *L. shawii* for 24 h. A negative control, cells without any treatment, was also used. According to the kit protocol, apoptosis/necrosis was monitored using the TACS Annexin V–FITC Apoptosis Detection Kit (R&D Systems, Minneapolis, MN, USA). The percentage of the apoptotic/necrotic cells was measured by flow cytometry analysis using a FACSCalibur flow cytometer (BD Biosciences, Becton Drive, Franklin Lakes, NJ, USA).

### 3.8. Antimicrobial Evaluations

#### 3.8.1. Test Organisms

*Staphylococcus aureus* (*S. aureus*) ATCC 29213, *Staphylococcus saprophyticus* (*S. saprophyticus*) ATCC 43867, *Streptococcus pyogenes* (*S. pyogenes*)-A ATCC 19615, *Streptococcus pneumoniae* (*S. pneumoniae*) ATCC 49619, *Enterococcus faecalis* (*E. faecalis*) ATCC 29212, *Bacillus cereus* (*B. cereus*) ATCC 10876, *Escherichia coli* (*E. coli*) ATCC 25922, *Klebsiella pneumoniae* (*K. pneumoniae*) ATCC 27736, *Pseudomonas aerugenosa* (*P. aerugenosa*) ATCC 9027, *Salmonella typhimurium* (*S. typhimurium*) ATCC 13311, *Shigella flexneri* (*S. flexneri*) ATCC 12022, *Proteus vulgaris* (*P. vulgaris*) ATCC 6380, *Proteus mirabilis* (*P. miribilis*) ATCC 29906, *Candida albicans* (*C. albicans*) ATCC 10231, and *Aspergillus niger* (*A. niger*) ATCC 6275 were used as the test organisms.

#### 3.8.2. Antimicrobial Activity

##### Preliminary Antimicrobial Activity

The preliminary antimicrobial activity evaluations of the four plants’ *L. shawii*, *A. articulata*, *R. vesicarius*, and *Z. spinosa* extracts were performed by the well-diffusion method [86,87]. The antimicrobial activity evaluation was conducted on modified tryptic soy-agar plates. The plant extracts were dissolved in sterile distilled water at a concentration of 100 mg/mL. The levofloxacin (70 µg/mL) and clotrimazole (2.5 mg/mL) were used as antibacterial and antifungal control antibiotics, respectively. First, the suspension of the test organisms (24–48 h old) was adjusted to a turbidity of 0.5 McFarland standard. Next, the 100 µL suspension of each test organism was independently dispensed on the agar plate, and using the sterile cotton swabs the suspensions were evenly distributed across the surfaces of the test plates. Next, the inoculated agar plates were punched, and wells were prepared using a sterile cork borer (06 mm diameter), and an 80 µL sample was used for each well. The inoculated plates were kept at 4–8 °C for 30 min and the samples were allowed to diffuse in the agar plates. Next, the inoculated plates were incubated at 35 ± 2 °C for 24 h for bacteria, and 30 ± 2 °C for 48–72 h for the fungi. Following incubation, the inhibitory zones’ diameters were measured on a millimeter scale. Each test was conducted in triplicate, and the findings were recorded in mean ± SD (standard deviation).

##### MIC and MBC

MIC was measured using the resazurin-based micro broth dilution method, whereas the MBC was measured using the spot inoculation method [86,87,88,89]. Two folds serial dilutions of the selected plant extract were prepared with starting concentration of 100 mg/mL with sterile distilled water, and subsequently, various concentrations of the plant extract (0.098–50 mg/mL) were prepared in sterile tryptic soy-broth (TSB). The prepared concentrations of the selected plant extracts were evaluated for their antimicrobial efficacy against selected pathogens. Levofloxacin (10 μg/mL), and clotrimazole (20 µg/mL) were used as control antibiotics.

### 3.9. Statistical Analysis

The results obtained from the preliminary antimicrobial screening of *Lycium shawii*, *Anabasis articulata*, *Rumex vesicarius*, and *Zilla spinosa* were analyzed to see whether the tested organisms have statistically different mean values of the antimicrobial activity. A one-way ANOVA test combined with Tukey’s analysis method (post hoc analysis) was conducted on SPSS version 20.0 SPSS (IBM, Armonk, NY, USA).

## 4. Conclusions

The study confirmed different phytochemicals representations and trace element concentrations in the four halophytic plants, *L. shawii*, *A. articulata*, *R. vesicarius*, and *Z. spinosa*, which are growing together in the arid area of central Saudi Arabia. The similarities in these plants’ biosynthetic ability to produce phenolics and flavonoids at varying concentrations compared to plants growing in normal, non-saline, non-desert environments have been recognized. The high concentrations of phenolics and flavonoids in these halophytic plants were likewise found to be associated with their higher anticancer and antimicrobial activities as compared to their non-halophytic counterparts. The higher levels of antioxidant potential, especially of the *L. shawii*, confirmed the importance of phenolics, and flavonoids contents as biologically active plant constituents. The higher presence of trace elements also supported the prevalent use of *L. shawii* as part of food and animal feeds, and their use in various biological, physiological, and symptomatic reliefs of various ailments by the local population, and the Bedouins. However, extensive pharmacological, bioassay-based activity localization in extracts/fractions, preclinical, and clinical studies are required to approve the plants’ safety for long-term, and proven-dose human consumption.

## Figures and Tables

**Table 1:** Trace elements contents of the four plants (µg/kg).

*Elements*	*Lycium shawii*	*Anabasis articulata*	*Rumex vesicarius*	*Zilla spinosa*
Fe	12.33 ± 4.16	24.33 ± 2.08	16.67 ± 2.31	93.47 ± 1.16
Cu	10.34 ± 2.11	13.67 ± 0.50	10.96 ± 0.93	10.44 ± 0.95
Mn	28.53 ± 0.42	39.1 ± 0.85	96.03 ± 1.04	34.8 ± 0.36
Co	15.67 ± 4.07	14.07 ± 1.31	18.0 ± 3.55	12.23 ± 2.07
Zn	12.93 ± 1.34	44.63 ± 0.40	109 ± 0.00	10.52 ± 1.10
Mg	1163 ± 9.17	1272 ± 18.52	1250 ± 20.0	1191 ± 10.14

**Table 2:** LC-MS result analyses of the four plants.

Sr	RT (min)	Observed Mass (m/z)	Calcd. Mass (m/z)	Ion	Molecular Formula	Identity *	Relative % of the Identified Compounds **			
							*R. vesicarius*	*L. shawii*	*A. articulata*	*Z. spinosa*
1	0.54	181.0721	182.0794	[M-H]-	Sorbitol	C_6_H_14_O_6_	0.0859	0.0358	0.0008	0.0013
2	0.55	341.1070	342.1138	[M-H]-	Hexose-based disaccharide	C_12_H_22_O_11_	2.7116	4.0265	0.1345	0.2199
3	0.55	683.2218	684.2291	[M-H]-	Galabiose	C_24_H_44_O_22_	0.0262	0.0818		
4	1.00	202.1069	203.1142	[M-H]-	L-Acetyl carnitine	C_9_H_17_NO_4_	0.0260	0.0053	0.0003	0.0006
5	2.21	165.0533	166.0606	[M-H]-	3-Phenyl lactic acid	C_9_H_10_O_3_		0.1023		0.0013
6	2.95	353.0859	354.0931	[M-H]-	Chlorogenic acid	C_16_H_18_O_9_	0.4393	0.0056		
7	4.72	387.1985	388.2058	[M-H]-	5-Methoxy-7,8-diprenylflavone	C_26_H_28_O_3_		0.0005	0.0007	0.0004
8	4.76	471.1875	472.1947	[M-H]-	Eugenol rutinoside	C_22_H_32_O_11_		0.0010	0.0014	0.0006
9	4.89	463.0886	464.0959	[M-H]-	Spiraeoside	C_21_H_20_O_12_	0.0005			
10	4.95	447.0890	448.0963	[M-H]-	Orientin	C_21_H_20_O_11_		0.0021	0.0003	0.0002
11	5.54	593.1488	594.1560	[M-H]-	Quercetin-3,7-dirhamnosyl	C_27_H_30_O_15_	0.0311	0.0118	0.0006	0.0011
12	5.60	609.1427	610.1500	[M-H]-	3-Gluco-7-rhamnosyl quercetin	C_27_H_30_O_16_	0.0050	1.3642	0.0069	0.0346
13	5.73	463.0861	464.0934	[M-H]-	Hyperoside	C_21_H_20_O_12_	0.2741	0.0087	0.0003	
14	5.92	447.0906	448.0978	[M-H]-	Luteolin 7-O-glucoside	C_21_H_20_O_11_	0.0054	0.1352		
15	6.32	577.1539	578.1625	[M-H]-	Isorhoifolin	C_27_H_30_O_14_	0.0406	0.0123		
16	6.36	593.1472	594.1545	[M-H]-	Kaempferol 3-neohesperidosid	C_27_H_30_O_15_	0.0008	1.5347	0.0328	0.2967
17	6.57	447.0918	448.0991	[M-H]-	Kaempferol-3-O-glucoside	C_21_H_20_O_11_	0.2531	0.0081		
18	6.81	285.0402	286.0475	[M-H]-	3,6,2’,4’-Tetrahydroxyflavone	C_15_H_10_O_6_		0.0053		
19	6.81	447.0935	448.1008	[M-H]-	Luteolin-4’-O-glucoside	C_21_H_20_O_11_	0.0010	0.0211		
20	6.82	431.0962	432.1035	[M-H]-	Vitexin	C_21_H_20_O_10_	0.0031	0.0351	0.0013	
21	6.90	431.0985	432.1058	[M-H]-	Isovitexin	C_21_H_20_O_10_	0.0006	0.0543	0.0002	
22	7.76	829.2216	830.2289	[M-H]-	Alatanin	C_39_H_42_O_20_	0.0052	0.0007		
23	7.77	289.1097	290.1169	[M-H]-	5-O-Methylvisamminol	C_16_H_18_O_5_		0.0307	0.8107	0.0879
24	8.06	312.1217	313.1290	[M-H]-	Acetylcaranine	C_18_H_19_NO_4_	0.0054	0.0011	0.0258	0.0033
25	8.59	301.0335	302.0407	[M-H]-	Quercetin	C_15_H_10_O_7_	0.9066	0.0009		
26	9.00	431.0985	432.1058	[M-H]-	Apigenin-7-O-glucoside	C_21_H_20_O_10_	0.0069	0.0003		
27	9.17	809.4290	810.4363	[M-H]-	Azukisaponin III	C_42_H_66_O_15_		0.4169		0.0582
28	9.24	315.0506	316.0579	[M-H]-	6-Methoxy luteolin	C_16_H_12_O_7_	0.5725	0.0041		
29	9.99	582.2586	583.2659	[M-H]-	Tricoumaroyl spermidine	C_34_H_37_N_3_O_6_	0.0116	0.3624	0.0034	0.0214
30	10.35	315.0508	316.0580	[M-H]-	Rhamnetin	C_16_H_12_O_7_	1.2297	0.0015		
31	11.70	329.2309	330.2382	[M-H]-	9,10,11-Trihydroxy-(12Z)-12- octadecenoic acid	C_18_H_34_O_5_	0.2259	0.0448	0.6680	0.3237
32	14.24	247.1339	248.1412	[M-H]-	3-Hydroxy-14-calamenoic acid	C_15_H_20_O_3_	5.1008	0.0380	0.0034	0.005
33	14.25	293.1737	294.1810	[M-H]-	Gingerol	C_17_H_26_O_4_	0.2933	0.3180	0.2920	0.3002
34	20.49	293.2101	294.2174	[M-H]-	Hydroxyoctadectrienoic acid	C_18_H_30_O_3_	0.0406	0.0096	0.0258	0.0806
35	25.68	253.2149	254.2222	[M-H]-	9-Hexadecenoic acid	C_16_H_30_O_2_	0.0819	0.1653	0.1796	0.0264
36	26.16	279.2304	280.23773	[M-H]-	Linoleic acid	C_18_H_32_O_2_	0.2374	0.4492	0.7048	2.0156
37	27.42	621.4395	622.4468	[M-H]-	Ginsenoside	C_36_H_62_O_8_	2.4514	0.2084		
38	27.91	255.2308	256.2381	[M-H]-	Palmitic acid	C_16_H_32_O_2_	1.5057	2.0933	2.8938	4.1485
39	28.23	281.2464	282.2536	[M-H]-	Oleic acid	C_18_H_34_O_2_	1.2639	1.5673	2.0126	3.0127
40	29.63	311.2242	312.2315	[M-H]-	Octadecenedioic acid	C_18_H_32_O_4_	2.6267	0.0293	0.0002	0.0011
41	29.72	575.4705	576.4778	[M-H]-	*cis*-Epoxy octadecenoate	C_36_H_64_O_5_	3.1036	0.0091		
42	29.96	283.2620	284.2693	[M-H]-	Stearic acid	C_18_H_36_O_2_	6.0177	8.1601	10.6431	11.1964
43	30.16	409.3085	410.3157	[M-H]-	γ-Tocotrienol	C_28_H_42_O_2_	0.4789	0.6317	0.7026	0.5591
44	30.30	423.4227	424.4299	[M-H]-	Octacosanoic acid	C_28_H_56_O_2_	0.0020		0.0003	0.0126
Total relative percentages of the identified compounds							30.07%	21.99%	19.15%	22.41%

** Compounds were tentatively identified; * relative % (percentages) of the occurrence levels of the identified compounds were calculated in comparison to the area of all the peaks in the LC-chromatogram for each plant.

**Table 3:** Quantitative analysis of the total phenolics, flavonoids, and antioxidant activity in mg/gm of the dried plants’ extracts.

Quantitative Tests Plants	TPC	TFC	TAA	FRAP	DPPH-SA	MCA
*Lycium shawii*	52.72 ± 3.17	13.01 ± 0.63	25.60 ± 4.61	56.68 ± 0.62	19.76 ± 0.04	21.84 ± 0.22
*Anabasis articulata*	21.13 ± 0.32	11.48 ± 1.52	12.43 ± 0.46	19.67 ± 0.40	7.15 ± 0.46	11.89 ± 0.31
*Zilla spinosa*	22.36 ± 0.67	7.29 ± 0.26	14.36 ± 0.38	23.68 ± 0.93	7.22 ± 0.13	13.32 ± 0.58
*Rumex vesicarius*	28.54 ± 1.13	12.64 ± 0.28	10.79 ± 0.46	33.09 ± 2.10	14.22 ± 0.29	13.01 ± 0.09

All the measurements were conducted in triplicate; mean and standard deviations were calculated. TPC, total phenolic contents calculated in mg/gm gallic acid equivalent; TFC, total flavonoid contents calculated in mg/gm quercetin equivalent; TAA, total antioxidant activity in mg Trolox equivalents per gm (gram) of the extract; FRAP, ferric reducing antioxidant power in mg Trolox equivalent per gm of the dry extract; DPPH-SA, 2,2-diphenyl-1-picrylhydrazyl-scavenging activity in mg Trolox equivalent per gm of the dry extract; MCA, metal chelating activity in mg; and EDTA, equivalents per gm of the extract.

**Table 4:** Antiproliferative IC_50_ values for the plants’ hydroalcoholic extracts.

Cell Lines	*Lycium shawii* (µg/mL)	*Anabasis articulata* (µg/mL)	*Rumex vesicarius* (µg/mL)	*Zilla spinosa* (µg/mL)
MCF-7, IC_50_ (95% CI)	194.5 (153.2 to 246.9)	2030 (944.7 to 4363)	1759 (661.2 to 4680)	1077 (661.7 to 1754)
K562, IC_50_ (95% CI)	464.9 (326.0 to 662.9)	2729 (1170 to 6366)	1319 (766.2 to 2270)	736.9 (475.6 to 1142)
PANC-1, IC_50_ (95% CI)	2619 (1246 to 5506)	998.5 (740.9 to 1346)	Not converged	Not converged
Fibroblast, IC_50_ (95% CI)	3109 (1225 to 7894)	3659 (1201 to 11,151)	3139 (1408 to 6999)	1888 (1105 to 3225)

**Table 5:** Annexin V-staining FACS analysis of the hydroalcoholic extract of *L. shawii*.

	(A) Media Untreated	(B) Half IC_50_	(C) IC_50_	(D) Double IC_50_
Viable Q3	64%	64.3%	36.2%	9.5%
Early apoptosis Q4	0.2%	1.2%	2.3%	0.4%
Late apoptosis Q2	12.8%	13.9%	33.4%	53.6%
Necrosis Q1	22.9%	20.6%	28.1%	36.5%

**Table 6:** Results of MIC and MBC of *Lycium shawii* (L), *Anabasis articulata* (A), and *Zilla spinosa* (Z) hydroalcoholic plant extracts and positive controls (PC).

Hydroalcoholic Plant Extracts	*B. cereus* ATCC 10876		*A. niger* ATCC 6275	
	MIC (mg/mL)	MBC (mg/mL)	MIC (mg/mL)	MBC (mg/mL)
*Lycium shawii*	12.5	25	-	-
*Anabasis articulata*	-	-	12.5	25
*Rumex vesicarius*	-	-	50	100

## Data Availability

Data are available in the main text and Supplementary File.

## Supplementary Materials

The following are available online at https://www.mdpi.com/article/10.3390/plants10102208/s1.
